# Maternal immune activation alters adult behavior, intestinal integrity, gut microbiota and the gut inflammation

**DOI:** 10.1002/brb3.2133

**Published:** 2021-04-01

**Authors:** Wenqiang Li, Mengxue Chen, Xia Feng, Meng Song, Minglong Shao, Yongfeng Yang, Luwen Zhang, Qing Liu, Luxian Lv, Xi Su

**Affiliations:** ^1^ Henan Mental Hospital The Second Affiliated Hospital of Xinxiang Medical University Xinxiang China; ^2^ Henan Key Lab of Biological Psychiatry of Xinxiang Medical University Xinxiang China; ^3^ International Joint Research Laboratory for Psychiatry and Neuroscience of Henan Xinxiang China; ^4^ Henan Province People's Hospital Zhengzhou China

**Keywords:** behavior, gut, inflammation, maternal immune activation, microbiota

## Abstract

**Background:**

Schizophrenia is characterized by several core behavioral features, in which the gastrointestinal symptoms are frequently reported. Maternal immune activation (MIA) has been developed in a rodent model to study neurodevelopmental disorders such as schizophrenia. However, the changes in the gut environment of MIA rats remain largely unknown.

**Methods:**

10 mg/kg of polyinosinic:polycytidylic acid (Poly I:C) on gestational day 9 was intravenously administered to rats to induce MIA in order to assess changes in behavior, the intestinal barrier and microbiota in offspring.

**Results:**

Maternal immune activation offspring shown increased anxiety as indicated by reduced exploration of central area in open field test and decreased exploration of open arms in elevated plus test. Cognitive impairment of MIA offspring was confirmed by reduced exploration of novel arm in Y maze test and deficiency of PPI. Intestinal muscle thickness became thinner and some specific microbial anomalies previously identified clinically were observed in MIA offspring. In addition, an increase of inflammatory responses was found in the gut of MIA offspring.

**Conclusions:**

Maternal immune activation alters behavior, intestinal integrity, gut microbiota and the gut inflammation in adult offspring.

## Graphical abstract

Maternal immune activation alters adult behavior. Maternal immune activation alters intestinal integrity, gut microbiota and the gut inflammation.

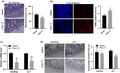

## 1 BACKGROUND

Schizophrenia is a common and severe neurodevelopmental disease that affects approximately 1% of the population. This disease is manifested by a disruption in cognition and emotion, along with other negative and positive symptoms. Although schizophrenia is characterized by core behavioral impairment, gastrointestinal (GI) symptoms are frequently reported (Nemani et al., 2015). A proportion of schizophrenic patients exhibit dysbiosis of the intestinal microbiota, with some demonstrate an increase in intestinal permeability (Akhondzadeh, 2019; Golofast & Vales, 2020; Nguyen et al., 2019).

The maternal immune activation (MIA) model of schizophrenia has been established based on several epidemiological studies (Brown et al., 2004; Mednick et al., 1988; Meyer & Feldon, 2010). In addition to the changes in neuromorphology, electrophysiology, neurochemistry and neuronal structure, the adult offspring of MIA rodent often exhibit behavioral alterations and other characteristics of schizophrenia (Meyer, 2014; Meyer et al., 2008). One of the most common technique for MIA induction is the supplementation of double‐stranded RNA analogue to the pregnant rodents (Meyer, 2014). Several reports have demonstrated that the offspring of pregnant dams exposed to polyinosinic:polycytidylic acid (poly I:C) display schizophrenia‐like behavior, including cognitive deficits, as well as exhibit neuroimmunological and brain morphological abnormalities (Bergdolt & Dunaevsky, 2019; Meyer, 2014; Murray et al., 2019). Hence, the poly I:C‐induced MIA model has emerged as a valuable research tool for the assessment of schizophrenia.

Commensal bacteria may induce various behavioral features such as emotional, social, and anxiety‐like behavior, thereby affecting the neuronal development and function of both mice (Cryan & Dinan, 2012) and humans (Tillisch et al., 2013). Gut microbiota are highly sensitive to early developmental environments, including MIA (Hsiao et al., 2013). More recently, it has been hypothesized intestinal microbiota can affect the host's brain development (Hsiao et al., 2013).

Schizophrenia is a complex mental disorder with unknown etiology. MIA is one of the most ideal models for studying neurodevelopment disorders (Brown & Meyer, 2018). Based on the recent emerging development of a gut‐microbiota‐brain axis, it is hypothesized that the behavioral features of schizophrenia may be related to GI deficits (Golofast & Vales, 2020). If analogous GI dysfunction exist in MIA rat model, it will be suitable to investigate the pathophysiology of the gut‐brain interaction of neuropsychiatry, and potentially suggesting the GI as a new target for psychotherapeutic selection. The aim of this study was to investigate whether MIA causes GI deficiency and schizophrenia‐like behaviors. Further investigations are needed to address questions related to the cause and relationship between the gut and the brain: do changes in the GI underlie the pathophysiology of schizophrenia or are they a result thereof?

## 2 METHODS

### 2.1 Animals

Sprague‐Dawley rats (8 weeks old) were supplied by Beijing Vital Rival. The rats were allowed to mate at 3 months old, and the occurrence of mating was verified by copulatory plugs. All rats were housed in ventilated cages at 22 ± 2°C and 50 ± 10% humidity, under a normal day/night cycle (lights on from 08:00 to 20:00). All animal handling and experimental procedures were conducted according to the National Institute of Health Guide for the Care and Use of Laboratory Animal. All animal sample collection protocols complied with the current laws of China. All animal procedures performed in this research were in accordance with the Laboratory Animal Ethics Committee of Henan Mental Hospital. (permit number: HMH. No20190806AEC004).

### 2.2 MIA during pregnancy

A timed‐mating procedure was employed for the construction of MIA model during rat pregnancy. On the 9th day after copulation, the mating dams were randomly assigned into two different groups (*n* = 8 for poly I:C administration group and *n* = 8 for saline administration group). Random numbers were generated using the standard = RAND() function in Microsoft Excel. The rat dams were intravenously injected with 10 mg/kg poly I:C (Sigma‐Aldrich) in saline, or an equal amount of saline solution according to previous study (Murray et al., 2019). Three hours after treatment, three rat dams of each group were randomly selected and anesthetized with 50 mg/kg sodium thiopental via intraperitoneal injection. Plasma samples were collected by cardiac puncture using sterile syringes from each rat for measuring the levels of interleukin (IL)‐1β, IL‐6 and tumor necrosis factor‐α (TNF‐α) through the use of enzyme‐linked immunosorbent assay (ELISA; R&D Systems).

### 2.3 Behavioral testing

Weaning of both pup groups was conducted on postnatal day 21. Previous study suggested sex‐specific effects, and particularly male vulnerability, following MIA (Hui et al., 2018; Meehan et al., 2017; Rahman et al., 2017), so male dams were selected for research and no more than two male rats from per litter are used in each group. Ten male rats from each group were randomly selected on postnatal day 60 for a typical behavioral assessment. The open‐field, elevated plus‐maze, and Y‐maze tests were performed with Spain pan Lab Smart version 3.0 software (RWD Life Science.) using an overhead video camera system to automate behavioral testing and provide unbiased data analyses. The open‐field and elevated plus‐maze tests were adopted to determine anxiety levels and locomotor activities, while Y‐maze test was used to evaluate spatial recognition memory. Then, sensorimotor gating was measured by the prepulse inhibition (PPI) analysis. All these behavioral tests were carried out between 08:00 and 18:00 hr. After each assessment, the apparatus were cleaned with 75% alcohol. All behavioral tests were performed as previously described (Hao et al., 2019).

### 2.4 Tissue collection

At adulthood, male rats were anesthetized with 50 mg/kg sodium thiopental via intraperitoneal injection 2 days after behavior testing (behavior testing began at postnatal day 60). Prefrontal cortex and colon tissues from each rat were subjected to mRNA and protein expression analyses. Rats were perfused with saline followed by 4% paraformaldehyde in PBS for hematoxylin and eosin, immunohistochemical and immunofluorescence staining. Stool samples were collected from male adult offspring prior to the start of any behavioral test. The samples were collected in the morning after transferring the animals to freshly disinfected cages.

### 2.5 RNA extraction and quantification

Total RNA was extracted with TRIzol (Invitrogen) by following the manufacturer's instructions. Quantitative real‐time polymerase chain reaction (qRT‐PCR) was conducted on an ABI StepOne RT‐PCR System (Applied Biosystems) using a QuantiTect SYBR Green RT‐PCR kit (Qiagen, 204245). GAPDH expression was stable between different MIA groups without difference and was chosen as the reference gene of MIA model (Hao et al., 2019). The copy numbers of target mRNAs were normalized to GAPDH mRNA levels in each sample. The primers for RT‐PCR are listed in the (Table S1).

### 2.6 Protein isolation and western blotting

Protein samples were isolated using radioimmunoprecipitation assay (RIPA) lysis buffer, followed by centrifugation at 12,000 ×*g* for 15 min at 4°C. After supernatant collection, the protein concentrations were evaluated by the Bio‐Rad Coomassie Blue protein assay (Bio‐Rad, Hemel Hempstead). Equal amounts of protein (40 μg) were separated on SDS‐PAGE, followed by Western blotting as previously described (Hao et al., 2019).

### 2.7 Antibodies and reagents

The primary antibodies used were as follows: anti‐IL‐1β (1:1,000; 16806–1‐AP; Proteintech), anti‐TNF‐α (1:1,000; 17590–1‐AP; Proteintech), and anti‐IL‐6 (1:1,000; 21865–1‐AP; Proteintech). The secondary horseradish peroxidase (HRP)‐conjugated antibodies used were as follows: goat anti‐rabbit HRP (1:5,000; 12–348) and goat anti‐mouse (1:5000; sc‐2005; Santa Cruz, Insight Biotechnology). Meanwhile, the immunofluorescent CY3 dye‐conjugated secondary antibody (1:300; Bost Biotech) was also used.

### 2.8 DNA extraction and analysis

Total DNA was isolated from fecal samples using a Fast DNA Stool Mini Kit (QIAGEN). Quantification of DNA yield was performed using a Nanodrop 2000 spectrophotometer. The amplification of six bacteria species (*Bacteroides* spp., *Bifidobacterium* spp., *Clostridium* coccoides, *Escherichia coli, Fusobacterium prausnitzii* and *Lactobacillus* spp.) was conducted on an ABI StepOne RT‐PCR System (Applied Biosystems) using a QuantiTect SYBR Green RT‐PCR kit (Qiagen, 204245). Bacterial copy numbers were calculated according to the obtained cycle thresholds (CT). The primers for Real‐Time PCR detection are listed in the (Table S1).

### 2.9 Statistical analysis

The data were analyzed using SPSS Statistics version 20.0 (SPSS Inc.) and were expressed as mean ± standard error of the mean (*SEM*). PPI dataset was analyzed using two‐way ANOVA (treatment and trials) with repeated measures. Independent student's *t*‐tests (two‐tailed) were used for other data analysis. A *p*‐value of <.05 was considered statistically significance. All experiments were performed at least three times and the representative data were presented.

## 3 RESULTS

### 3.1 Levels of cytokines in MIA rat model

**FIGURE 1:**
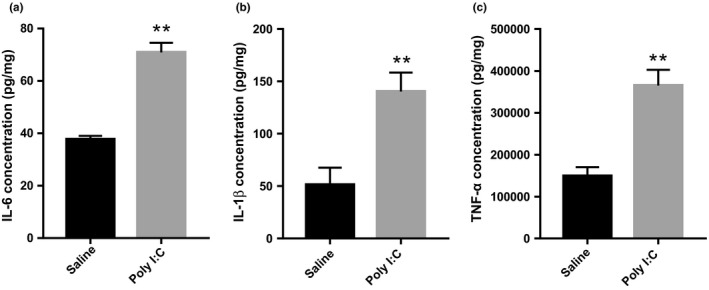
Inflammatory cytokine levels in pregnant rats exposed to Poly I:C or saline. The bar graph shows maternal IL‐6 (a), IL‐1β (b), and TNF‐α (c) blood levels measured 3 hr post 10 mg/kg Poly I:C or saline administration. Independent Student's *t*‐test (two‐tailed) was performed for statistical analysis. Results are shown as the mean ± *SEM* (*n* = 3 per group). *p <.05, **p <.01. The detection was repeated independently for three times

After administrating 10 mg/kg poly I:C to the pregnant rats, the inflammatory responses were assessed 3 hr following injection. Figure 1 demonstrates the levels of IL‐6 (*t* = 8.634, *df*=4, *p* =.0010), IL‐1β (*t* = 6.312, *df*=4, *p* =.0032), and TNF‐α (*t* = 5.046, *df*=4, *p* =.0073) in pregnant rats were upregulated in MIA‐induced rats compared to saline controls, confirming the activation of maternal immunity by poly I:C.

### 3.2 MIA leads to exacerbated anxiety in offspring

**FIGURE 2:**
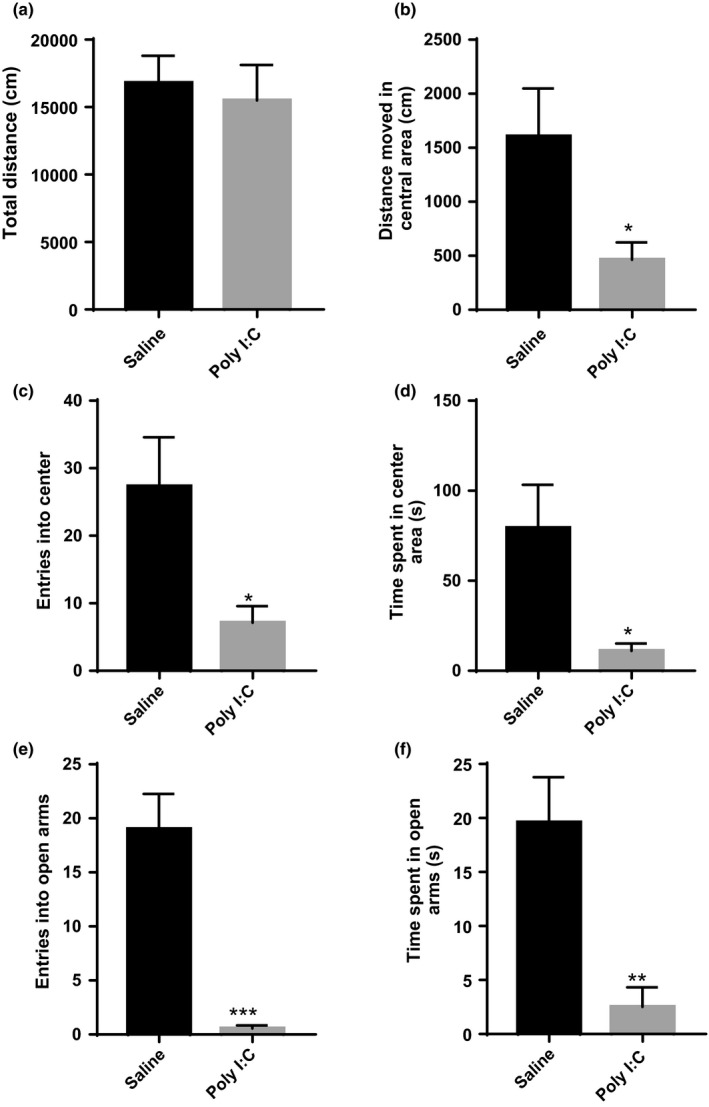
Prenatal immune activation induces anxiety behaviors in offspring. (a–d) The open field test. Total distance traveled in the open field arena (a), distance moved in the center arena (b), entries into the center arena (c) and time spent in the center arena (d). (e and f) The elevated plus maze test. Entries into the open arms (e) and time spent in open arms (f); *n* = 10 for each group. Independent Student's *t*‐test (two‐tailed) was performed for statistical analysis. Data are shown as mean ± *SEM*. *p <.05, **p <.01

Open‐field and elevated plus‐maze tests were conducted to evaluate the anxiety status of MIA offspring. During the exposure to an open‐field arena for 30 min, MIA offspring exhibited a decrease in distance moved within the central area (*t* = 2.419, *df*=18, *p* =.0264), although no differences were observed in distances moved within the whole field (Figure 2a,b). In addition, the animal entry into central areas (*t* = 2.625, *df*=18, *p* =.0172) and time spent in central areas (*t* = 2.795, *df*=18, *p* =.0120) were significantly decreased in MIA group compared to saline control group (Figure 2c,d). For the elevated plus‐maze test, prenatal poly I:C exposure reduced the percentage of time spent in the open arms (*t* = 5.330, *df*=18, *p* <.0001), with fewer entries into the open arms (*t* = 3.605, *df*=18, *p* =.0022) (Figure 2e,f). These findings reveal that MIA offspring display certain anxiety‐related behaviors.

### 3.3 MIA leads to cognitive impairment in offspring

**FIGURE 3:**
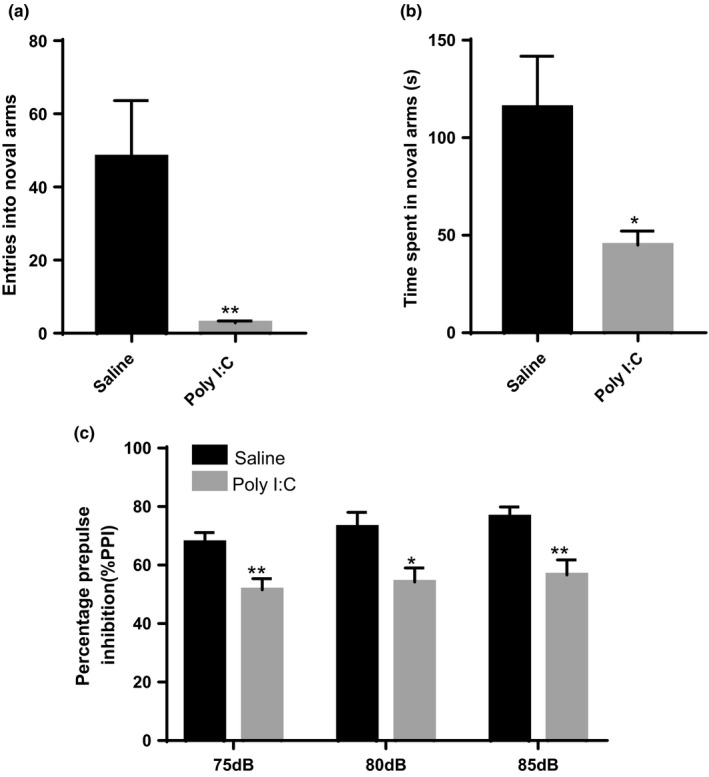
Prenatal immune activation induces cognitive impairment in offspring. (a and b) The Y‐maze test. Entries into the novel arm (a) and time spent in the novel arm (b) were analyzed. (c) % PPI performance at 75, 80, and 85 dB; *n* = 10 for each group. Independent Student's *t* test (two‐tailed) was performed for statistical analysis in (a and b); PPI dataset (c) was analyzed using Two‐way ANOVA (treatment and trials) with repeated measures. Data are mean ± *SEM*. *p <.05, **p <.01

To examine memory impairment, Y‐maze assessment was carried out. Over the course of a 5‐min period, poly I:C treatment groups showed fewer entries into the novel arm (*t* = 2.936, *df*=18, *p* =.0088) and decreased the percentage of time spent in the novel arm (*t* = 2.190, *df*=18, *p* =.0448), when compared with saline treated groups (Figure 3a,b).

Further, auditory sensory gating deficit was assessed by PPI test. Compared to control group, the percentage PPI at the prepulse intensities 75, 80, and 85 dB was markedly reduced in MIA rat offspring (Figure 3c).

Altogether, these behavioral findings indicate that MIA can induce schizophrenia‐like behavior in rat offspring.

### 3.4 MIA induces intestinal impairment in offspring

**FIGURE 4:**
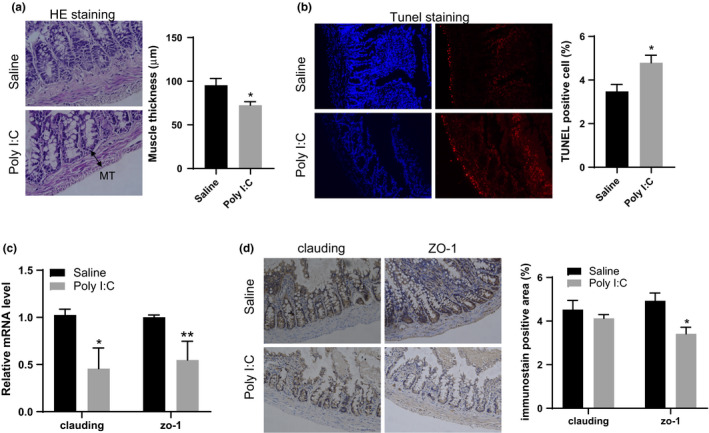
The effects of MIA on the intestinal epithelial barrier in offspring. (a) Representative images of HE staining of colon from MIA offspring were shown; Muscle thickness was analyzed from ten different regions of each sample (*n* = 3 for each group). MT: muscle thickness. (b) Representative images of TUNEL staining of colon from MIA offspring were shown and the percentage of TUNEL positive cell was calculated (*n* = 3 for each group). (c) The mRNA expression of the tight junction markers, clauding and ZO‐1 were detected in colon from MIA and control offspring (*n* = 6 for each group). (d) Representative images of IHC staining of claudin and ZO‐1 from MIA and control colon offspring were presented, and positive area of immunostain was calculated (*n* = 3 for each group). Independent Student's *t*‐test (two‐tailed) was performed for statistical analysis. All data are expressed as mean ± *SEM*. *p <.05, **p <.01. All experiments were repeated independently for three times

Subsets of schizophrenia patients are found to exhibit GI abnormalities, including leaky gut syndrome. Therefore, we further determine whether MIA can alter the pathophysiology of the intestines. It was found that the adult MIA offspring with schizophrenia changes displayed significant colon deficits in gut barrier integrity. MIA offspring exhibited hallmark distortions of gut morphology (*t* = 5.623, *df*=58, *p* <.0001), as indicated by the histological examination of HE‐stained sections (Figure 4a). Moreover, severe cellular injury was also observed in the colons of adult MIA offspring, as revealed by TUNEL staining (*t* = 2.804, *df*=4, *p* =.0486), an indicative of marked DNA damage (Figure 4b). Consistent with the defects in intestinal barrier integrity, the levels of the tight junction markers (i.e., ZO‐1 and CLDN) were reduced in the colon tissue of adult MIA offspring, as indicated by RT‐PCR (clauding: *t* = 3.190, *df*=10, *p* =.0110; zo‐1: *t* = 3.293, *df*=10, *p* =.0081)(Figure 4c) and immunohistochemical staining (zo‐1: *t* = 3.277, *df*=4, *p* =.0306)(Figure 4d). These observations suggest that intestinal barrier integrity is impaired in these animals.

### 3.5 MIA alters gut microbiota composition

**FIGURE 5:**
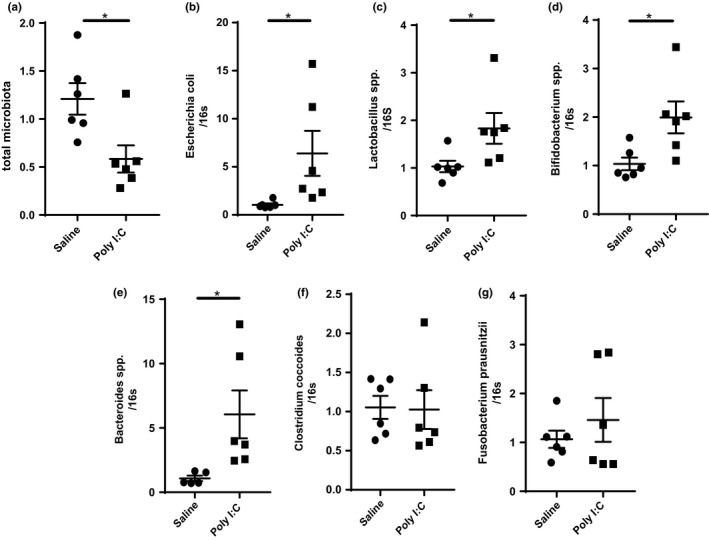
Specific fecal bacteria measurements in MIA and control offspring. Total microbiota count (a) and the proportion of *E. coli* (b), *Lactobacillus* spp. (c), *Bifidobacterium* spp. (d), *Bacteroides* spp. (e), *Clostridium* coccoides group (f) and *Fusobacterium prausnitzii* (g) were analyzed and shown as the mean ± *SEM*, *n* = 6 per group. Independent Student's *t*‐test (two‐tailed) was performed for statistical analysis. *p <.05, **p <.01. The detection was repeated independently for three times

Intestinal bacteria play important roles in maintaining intestinal homeostasis and function. Abnormal features associated with dysbiotic microbiome, such as altered bacterial community composition and content, have been observed in schizophrenia patients. To assess whether MIA can induce microbiota alteration, we examined several microorganisms that have been differentially reported in patients with schizophrenia. As illustrated in Figure 5a, the total number bacteria was significantly reduced in MIA offspring, as assessed by 16s rRNA amplification (*t* = 2.882, *df*=10, *p* =.0163). Moreover, the offspring exposed to MIA displayed significantly higher levels of *E. coli* (*t* = 2.279, *df*=10, *p* =.0459; Figure 5b), *Lactobacillus* spp. (*t* = 2.534, *df*=10, *p* =.0420; Figure 5c), *Bifidobacterium* spp. (*t* = 2.707, *df*=10, *p* =.0220; Figure 5d), and *Bacteroides* spp. (*t* = 2.406, *df*=10, *p* =.0395; Figure 5e) when compared with control animals. No significant changes were observed for the fecal *Clostridium* coccoides group (Figure 5f) and *Fusobacterium prausnitzii* spp. (Figure 5g) in MIA offspring when compared with control animals. These results indicate that common gut microbiota are remarkably altered in MIA offspring.

### 3.6 MIA induces intestinal inflammation in MIA offspring

**FIGURE 6:**
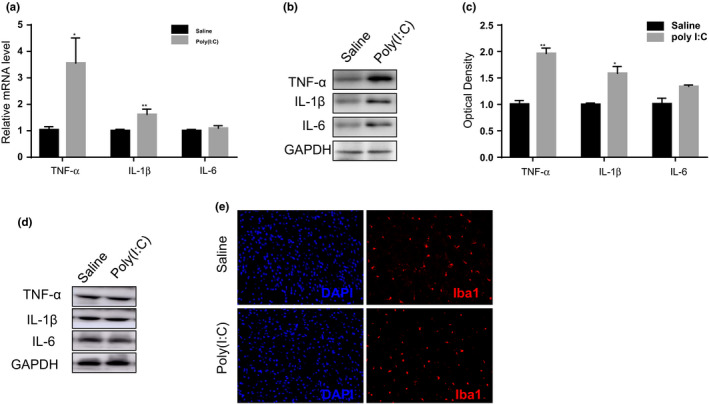
The inflammatory response in MIA and control offspring. (a–c) Maternal Poly I:C treatment induced gut inflammatory responses in offspring as characterized by increased mRNA and protein expression of the pro‐inflammatory cytokines TNF‐α and IL‐1β in the colon as indicated by real‐time PCR (a) and western blotting (b and c), with no significant changes was found in IL‐6 expression (a–c). (d and e) Inflammatory cytokine responses in prefrontal cortex of MIA and control offspring. The protein expression of the pro‐inflammatory cytokines IL‐1β, IL‐6, and TNF‐α in the prefrontal cortex of experimental animals as indicated by western blotting (d). Iba1 staining in the prefrontal cortex of MIA and control offspring (e). *n* = 6 for mRNA and protein expression and *n* = 3 for immunofluorescence staining for each group. Independent Student's *t*‐test (two‐tailed) was performed for statistical analysis. All data are expressed as the mean ± *SEM*. *p <.05, **p <.01. All experiments were repeated independently for three times

Intestinal permeability is often associated with the changes in immune responses. Notably, the colon tissue of adult MIA offspring displayed elevated mRNA (Figure 6a) and protein expression (Figure 6b,c) levels for IL‐1β and TNF‐α, while no change was observed for IL‐6 expression. Immune responses in the prefrontal cortex of MIA offspring were also detected (Figure 6d,e). The changes in intestinal cytokines were not accompanied with immune activation in the prefrontal cortex, as assessed by cytokine detection in the prefrontal cortex (Figure 6d). Through the use of microglia marker Iba1, no significant difference in prefrontal cortex staining was observed between MIA offspring and control animals (Figure 6e). This observation suggests the absence of inflammation in the prefrontal cortex of MIA offspring.

Collectively, it was observed that the adult offspring of MIA dams exhibited increased intestinal permeability and abnormally expressed levels of cytokines, which are consistent with the characteristics of human schizophrenia.

## 4 DISCUSSION

Although the underlying etiology remains unclear, there is substantial evidence of an association between psychiatric disorders and GI disease (Stefano et al., 2018; Van Oudenhove et al., 2010). Animal models are ideal tools for elucidating the mechanisms of human diseases. This study aimed to determine whether the well‐characterized MIA model can produce an intestinal environment imbalance concomitant with behavioral abnormalities. Our results indicated that the MIA rat model not only displays well‐documented behavioral abnormalities, but also exhibits intestinal integrity disruption, microbial imbalances, and gut inflammation. These data suggest that this model may be a useful tool for elucidating the biological mechanisms associated with GI disorders in psychiatric patients.

Accumulating evidence suggests that exposure to MIA during pregnancy increases the risk of neuropsychiatric diseases, including autism spectrum disorder and schizophrenia (Minakova & Warner, 2018). Epidemiological findings suggest that maternal infection during early‐to‐mid human pregnancy is more likely to be associated with long‐term developmental brain and behavioral abnormality in the offspring (Brown et al., 2004; Mednick et al., 1988). Different time periods of poly (I:C) exposure can lead to different emergent behaviors and neuropsychiatric changes throughout the offspring life, and infection and related inflammation in the second trimester of gestation can result in long‐lasting consequences with regard to brain development and behavior (Meyer, Nyffeler, et al., 2006). However, few reports have suggested that prenatal immune activation during early pregnancy can induce neuropsychiatric disease (Hao et al., 2019; Meyer et al., 2006; Rahman et al., 2017). Previous in vivo findings have indicated that prenatal immune activation during early pregnancy, especially on gestation day 9, can induce abnormal behavioral features, including working memory impairment and PPI deficit (Hao et al., 2019). Our results confirmed that early immune activation led to schizophrenia‐like behavioral abnormalities in rats. Although psychiatric disorders are typically characterized by core behavioral impairment, GI abnormalities are frequently reported (Sharon et al., 2016). A growing body of literature has linked neurobehavioral symptoms to gut–brain axis dysfunction (Hsiao et al., 2013; Mayer, 2011; Mayer et al., 2014). Subsets of patients exhibit dysbiosis of the gut microbiota, and some demonstrate increased intestinal permeability (Sharon et al., 2016). Recently, several elements of autism spectrum disorder‐associated and schizophrenia‐associated gut–brain axis dysfunction were reproduced in the MIA poly (I:C) model of C57BL6/J mice (Hsiao et al., 2013). MIA and maternal separation could trigger intestinal abnormalities such as increased intestinal permeability and visceral pain, along with certain behavioral features (Amini‐Khoei et al., 2019; Labouesse et al., 2015; O'Mahony et al., 2011). Additionally, the animals exposed to early‐life immune activation or psychological stress also exhibited intestinal dysbiosis (Amini‐Khoei et al., 2019; Rincel et al., 2019).

Currently, there is a dearth of studies on schizophrenia and its associated microbiota. Our study examined the changes in specific types of bacteria, such as Bacteroides spp., Bifidobacterium spp., Clostridium coccoides group, E. coli, and Lactobacillus spp., in adult MIA rat offspring, when compared to control group. Our MIA rat model showed that some of these differences were consistent with the clinical case control study of schizophrenia. Higher incidences of lactic‐acid bacteria in schizophrenia have been observed in the microbiota composition of oropharynx, when compared with controls (Castro‐Nallar et al., 2015). Additionally, at the family level, Lactobacillaceae was increased in first‐episode psychosis patients compared to controls (Schwarz et al., 2017).

A balanced intestinal environment with the microbiota plays an essential role in the health status of the host, by exerting beneficial effects on neurodevelopment and behavior phenotypes (Sharon et al., 2016). In a major subset of patients, intestinal symptoms arose before the onset of a mood disorder, suggesting that primary gut disturbance may serve as an underlying driver in some patient subgroups (Sharon et al., 2016). Another clue confirming the gut microbiota regulates behaviors comes from studies of germ‐free mice (Chu et al., 2019). These mice tend to have hyperactivity disorder and risk‐taking behaviors as well as memory and learning impairment when compared to conventional (specific‐pathogen‐free) mice (Clarke et al., 2013; Gareau et al., 2011; Diaz Heijtz et al., 2011; Neufeld et al., 2011; Sharon et al., 2016). Previous findings have indicated that the gut microbiota can affect the expression levels of genes in the medial prefrontal cortex (mPFC) (Hoban et al., 2016, 2017). The mPFC can regulate specific emotional behaviors and mediate the effects of early‐life stresses (Hao et al., 2019). Intestinal alterations, especially gut microbiota dysbiosis and loss of barrier functions, can affect brain development and permanently diminish gut‐brain communications (Meyer, 2014).

Long‐range interactions between the brain and the gut microbiota have been reported (Bravo et al., 2011; Ochoa‐Reparaz et al., 2010). A recent study reported GI barrier impairment and microbiota alteration in the MIA mice (Hsiao et al., 2013). Administration of *Bacteroides fragilis* to MIA offspring could improve gut permeability and microbial composition, as well as prevent abnormal communicative, stereotyped, sensorimotor, and anxiety‐like behaviors (Hsiao et al., 2013). Numerous studies, using MIA or maternal separation models, have demonstrated that probiotic treatment and fecal transplantation can mediate brain function and behavioral features (Desbonnet et al., 2010; Hsiao et al., 2013). Some studies have suggested probiotics are effective in treating the emotional symptoms of chronic fatigue syndrome and psychological distress in humans (Messaoudi et al., 2011; Rao et al., 2009).

The potential roles of microbiota in the etiology of psychiatric disease should be tested. Growth studies have suggested that most of the effects of microbiota on brain are regulated by microbial metabolites. Kynurenine (Kyn) accumulation in the brain has been associated with depression and schizophrenia (Muller & Schwarz, 2007); gut microbiota regulation of tryptophan (Trp) metabolism across three metabolic pathways can lead to the formation of serotonin, Kyn and indole derivatives (Cervenka et al., 2017). Although the blood‐brain barrier is highly selective, Trp and Kyn derivatives can pass through this barrier and elicit noticeable effects on neurotransmitter function (Cervenka et al., 2017). Kynurenic acid and Quin, produced directly from Kyn, affect the brain differently by binding to glutamate receptors (e.g., N‐methyl‐D‐aspartate receptor), which is essential for memory function (Cervenka et al., 2017). Short‐chain fatty acids (SCFAs), which are important bacterial fermentation products, drive microglia maturation to fulfil the requirement for microglia maintenance (Erny et al., 2015). Interestingly, decreased SCFA levels in the feces of Parkinson's disease patients were recently reported (Unger et al., 2016). Previous research has suggested that treatment with 4‐ethylphenylsulfate to naive wild‐type mice can induce anxiety‐like behaviors, as similar to those observed in MIA offspring (Hsiao et al., 2013).

Neuroinflammation plays an essential role in the pathophysiology of neurodegenerative disease (Cappellano et al., 2013; Severance et al., 2013). Proinflammatory cytokines are secreted from the brain and peripheral neurons (McCoy & Tansey, 2008). The passage of microbially associated molecular patterns from the intestine to the brain can result in low inflammation levels (Pal et al., 2015). Such persistent proinflammatory signaling is associated with the severity of neurodegenerative disease (Cappellano et al., 2013). However, our results indicated that no obvious changes in the levels of proinflammatory cytokines were found in the prefrontal cortex of MIA offspring, although increased proinflammatory cytokine levels were detected in the intestines. Microglia are the major sources of cytokine and chemokine expression in the brain parenchyma; however, our results showed no obvious microglial anomalies in the prefrontal cortex of MIA offspring. Recent work has reported that multiple microglial and astrocyte markers remained unchanged in the midbrains of MIA offspring, as detected by Western blotting, even though their mRNA levels were increased (Purves‐Tyson et al., 2019). These authors explained that RNA detection may be more sensitive for immune activation (Purves‐Tyson et al., 2019), which could also support our divergent results. Another study indicated Poly I:C significantly increased microglial activation at PND21 in male hippocampi, which support the hypothesis of neuroinflammation of MIA (Murray et al., 2019). The brain inflammatory state of MIA offspring may vary in different brain regions or stage of development.

This study was limited as it was an observational study, and the association between intestinal changes and behavioral deficits was not determined. It is unclear whether gut microbiota can confer risk to neuropsychiatric disorders or is a consequence of pathological processes. Therefore, future investigation is needed to understand the intricate and precise interactions between a host and its associated microbial communities. Another limitation of this study is that only male rats were analyzed and future research need to avoid sex bias.

## CONFLICT OF INTEREST

The authors declare that they have no competing interests.

### PEER REVIEW

The peer review history for this article is available at https://publons.com/publon/10.1002/brb3.2133.

## Supporting information

Tab S1

## Data Availability

The datasets used and/or analyzed during the current study are available from the corresponding authors upon reasonable request.
