# Supplementary material for: Maternal immune activation alters adult behavior, intestinal integrity, gut microbiota and the gut inflammation
Source: Brain Behav. 2021 Apr 1;11(5):e02133. doi: 10.1002/brb3.2133 (PMC8119836; doi:10.1002/brb3.2133)
Supplement: Supplementary file 1 — Tab S1 [file BRB3-11-e02133-s001.pptx]

## Slide 1
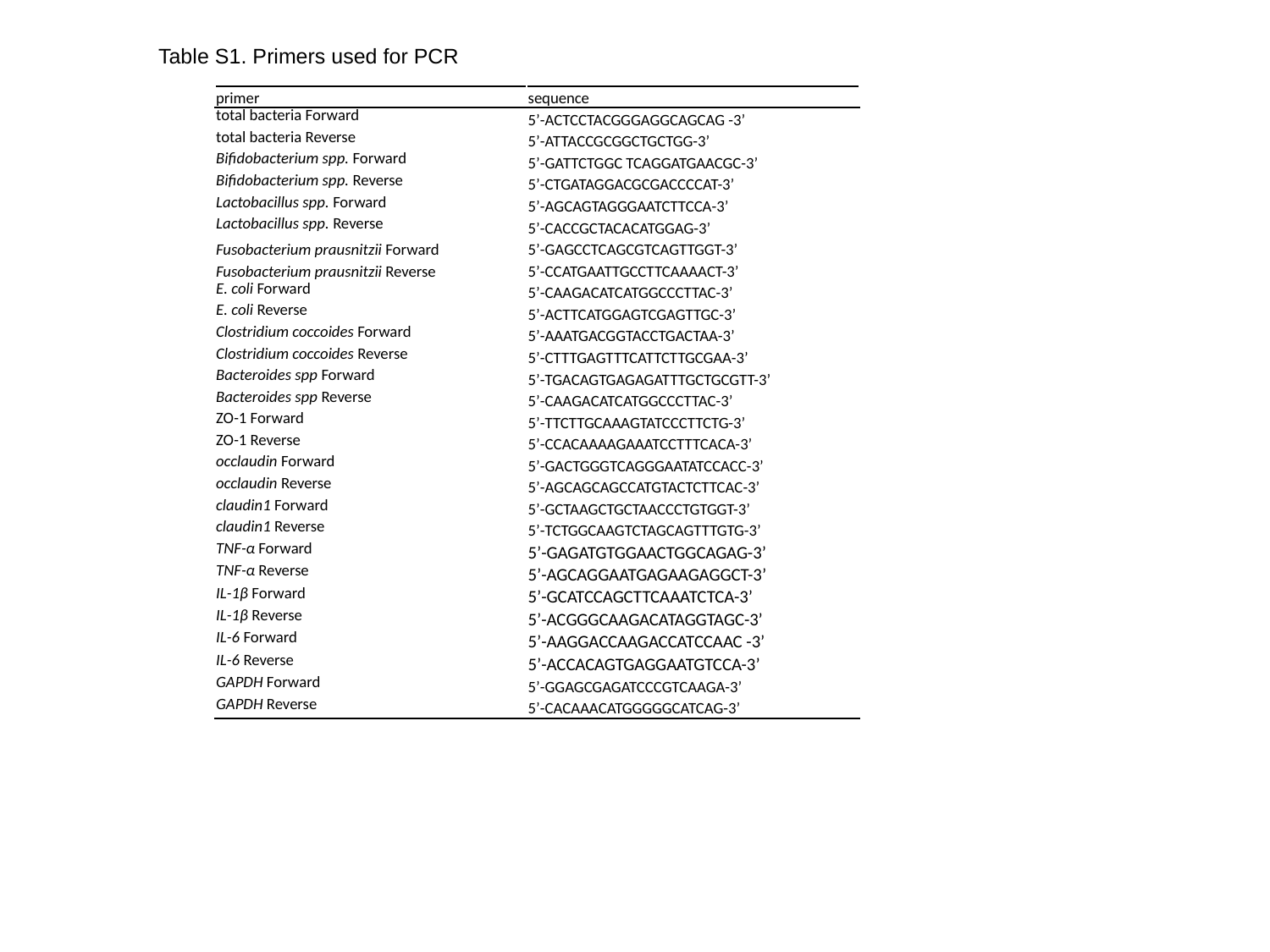

Table S1. Primers used for PCR
| primer | sequence |
| --- | --- |
| total bacteria Forward | 5’-ACTCCTACGGGAGGCAGCAG -3’ |
| total bacteria Reverse | 5’-ATTACCGCGGCTGCTGG-3’ |
| Bifidobacterium spp. Forward | 5’-GATTCTGGC TCAGGATGAACGC-3’ |
| Bifidobacterium spp. Reverse | 5’-CTGATAGGACGCGACCCCAT-3’ |
| Lactobacillus spp. Forward | 5’-AGCAGTAGGGAATCTTCCA-3’ |
| Lactobacillus spp. Reverse | 5’-CACCGCTACACATGGAG-3’ |
| Fusobacterium prausnitzii Forward | 5’-GAGCCTCAGCGTCAGTTGGT-3’ |
| Fusobacterium prausnitzii Reverse | 5’-CCATGAATTGCCTTCAAAACT-3’ |
| E. coli Forward | 5’-CAAGACATCATGGCCCTTAC-3’ |
| E. coli Reverse | 5’-ACTTCATGGAGTCGAGTTGC-3’ |
| Clostridium coccoides Forward | 5’-AAATGACGGTACCTGACTAA-3’ |
| Clostridium coccoides Reverse | 5’-CTTTGAGTTTCATTCTTGCGAA-3’ |
| Bacteroides spp Forward | 5’-TGACAGTGAGAGATTTGCTGCGTT-3’ |
| Bacteroides spp Reverse | 5’-CAAGACATCATGGCCCTTAC-3’ |
| ZO-1 Forward | 5’-TTCTTGCAAAGTATCCCTTCTG-3’ |
| ZO-1 Reverse | 5’-CCACAAAAGAAATCCTTTCACA-3’ |
| occlaudin Forward | 5’-GACTGGGTCAGGGAATATCCACC-3’ |
| occlaudin Reverse | 5’-AGCAGCAGCCATGTACTCTTCAC-3’ |
| claudin1 Forward | 5’-GCTAAGCTGCTAACCCTGTGGT-3’ |
| claudin1 Reverse | 5’-TCTGGCAAGTCTAGCAGTTTGTG-3’ |
| TNF-α Forward | 5’-GAGATGTGGAACTGGCAGAG-3’ |
| TNF-α Reverse | 5’-AGCAGGAATGAGAAGAGGCT-3’ |
| IL-1β Forward | 5’-GCATCCAGCTTCAAATCTCA-3’ |
| IL-1β Reverse | 5’-ACGGGCAAGACATAGGTAGC-3’ |
| IL-6 Forward | 5’-AAGGACCAAGACCATCCAAC -3’ |
| IL-6 Reverse | 5’-ACCACAGTGAGGAATGTCCA-3’ |
| GAPDH Forward | 5’-GGAGCGAGATCCCGTCAAGA-3’ |
| GAPDH Reverse | 5’-CACAAACATGGGGGCATCAG-3’ |
